# ‘Placebo’ is a misnomer in clinical care: deceptive inert-use must be distinguished from contextual neuropsychological effects

**DOI:** 10.31744/einstein_journal/2026RW2157

**Published:** 2026-09-18

**Authors:** José Nunes de Alencar, Francisca Rego, Rui Nunes

**Affiliations:** 1 Faculdade de Medicina Universidade do Porto Porto Portugal Faculdade de Medicina. Universidade do Porto, Porto, Portugal.; 2 Instituto Dante Pazzanese de Cardiologia São Paulo SP Brazil Instituto Dante Pazzanese de Cardiologia, São Paulo, SP, Brazil.

**Keywords:** Placebos, Ethics, Neuropsychology

## Abstract

The term placebo effect collapses distinct phenomena—natural history, regression to the mean, co-interventions, observation effects, and genuine context-dependent neuropsychological responses—into a single, misleading label. To clarify where causal attribution goes wrong, we propose testable terminology that separates inert-substance effects from contextual determinants of outcome, and outline designs that estimate context effects without deception. We synthesize evidence that clinical context (expectation, meaning, interactional cues, and ritual) modulates symptoms and some physiologic measures, while emphasizing that within-arm change does not identify a causal effect of an inert substance. We recommend reserving the term placebo for its methodological role in randomized trials and using contextual effects for outcomes driven by psychosocial and neurobiological processes. We define deceptive inert-substance use as ethically unacceptable and distinguish it from open-label strategies that transparently leverage context. We summarize study designs—open-hidden administration, no-treatment comparators, factorial context manipulations, and balanced-placebo frameworks—that can estimate context effects and specify typical biases (e.g., adherence bias) and adjustments. Clinicians should deliberately harness context through communication quality, empathic framing and ritual clarity without mislabeling inert substances as therapeutic. Researchers should prespecify estimands for context, report between-arm contrasts, and avoid interpreting control-arm changes as “placebo effects.” Replacing an imprecise placebo effect narrative with a causal and ethical framework—contextual effects plus and explicit prohibition of deceptive inert- substance use—aligns clinical practice with evidence and supports patient trust.

## INTRODUCTION

The concept of the placebo effect is often cited as a self-evident truth within both clinical and public discourse, purportedly illustrating a clear and simple phenomenon of patient improvement due to belief in a treatment, rather than the treatment’s inherent therapeutic efficacy. It is estimated that approximately 40% of prescriptions function as a placebo.^(1,2)^ However, the reality of the placebo effect is far from straightforward. The medical community is engaged in an ongoing debate not only about the existence of the placebo effect but also about its precise definition and implications. ^(3)^ Such misunderstandings are not merely academic debates; they have tangible impacts on public perception and healthcare practices.^(4)^ The oversimplification of the placebo effect allows for its misapplication as a means of validating therapies that lack scientific support, often championed by proponents of so-called alternative medicine.^(5-9)^

The inception of placebo research can be traced back to the mid-20th century, notably marked by Beecher’s 1955 publication, “The Powerful Placebo,”^(10)^ in which he quantitatively assessed the placebo effects across various diseases. In a review of 15 studies, Beecher asserted that 35% of patients experienced symptom relief solely due to placebo, a claim that has significantly shaped the debate on the use of placebo in clinical practice. This historical perspective underlines a tradition of placebo research, that has evolved to suggest the omnipresence of placebo effects in almost all medical conditions, and some current estimates even exceed Beecher’s initial findings.

Early studies by researchers such as Wolf et al.^(11)^ and Lasagna et al^(12)^ echoed Beecher’s observations, reporting substantial placebo effects across a range of clinical outcomes, both objective and subjective. Placebo effects have been documented in conditions such as asthma, hypertension, and survival after myocardial infarction,^(13)^ further cementing the role of the placebo effect in medical research and treatment paradigms.^(14)^

The central question of this article is how changes observed after administering an inert substance should be conceptualized and named so that causal claims are valid and the clinical use of context remains ethical and nondeceptive. To address this question, we pursue three aims. First, we explain why the term placebo effect misleads causal inference by collapsing natural history, regression to the mean, observation effects, co-interventions, and genuine context-dependent responses into a single label. Second, we propose a testable nomenclature that reserves placebo for the methodological role of inert comparators in randomized controlled trials (RCTs) and uses contextual (neuropsychological) effects to describe outcomes driven by expectations, meaning, and therapeutic ritual. Third, we outline study designs capable of estimating contextual effects without deception and discuss the ethical implications for clinical practice and research reporting.

## NEUROSCIENCE OF PLACEBO

In the realm of neuroscience, the study of placebos transcends mere clinical outcomes, offering a profound exploration of how the brain processes contextual information to influence physiology and treatment effects.^(15)^ This understanding hinges on recognizing both the external and internal contexts in which treatments are delivered. Externally, the clinical setting envelops the patient with a myriad of cues, from the physical environment to thesocial interactions, such as the presence of healthcare professionals. Internally, a patient’s brain is a hub of activity involving memories, emotions, and expectations, all of which contribute to the interpretation of these cues and the treatment context.^(16-18)^ This integration of external and internal contexts forms the crux of what is often perceived as the placebo effect, highlighting its foundation in the patient’s neurological response to the surrounding treatment framework.

Moreover, neuroscientific research has shown that these contextual elements can significantly modulate physiological outcomes through mechanisms rooted in the brain’s ability to integrate and respond to environmental cues. Techniques such as functional MRI (fMRI) and positron emission tomography (PET) have illuminated how areas like the prefrontal cortex engage in this complex processing of contextual information.^(19)^ Studies focusing on placebo effects in pain, for example, have revealed that placebo treatments can activate brain systems involved in analgesia and hyperalgesia, illustrating the brain’s integral role in mediating such effects.^(20-22)^

In addition, the impact of placebos extends to autonomic and neuroendocrine responses, governed by higher brain regions that respond dynamically to psychological contexts. Neuroimaging evidence supports that placebo interventions can modulate these systems, influencing outcomes in chronic conditions like Parkinson’s disease^(23)^ and depression.^(24)^ For instance, in the amygdala, placebo treatments are known to increase endogenous opioid responses—this modulation of neurochemical activity is a testament to the brain’s adaptability to psychological nuances introduced by placebo contexts.

## WHY “PLACEBO EFFECT” MISLEADS CAUSAL INFERENCE

One of the earliest definitions posited of a placebo states that it is any therapy or component of therapy that is deliberately used for its non-specific, psychological, or psychophysiological effect, or that is used for its presumed specific effect but is without specific activity for the condition being treated.^(25)^ This is a problematic concept because scientists providers and patients might hold different views on whether an intervention should be considered a placebo (Table 1).^(26)^

**Table 1:** Clinical scenarios and related views of providers, patients, and scientists regarding whether the intervention provided should be considered a placebo

Scenario	Provider view	Patient view	Shapiro’s definition
Saline injection in a patient in pain	Placebo	Specific therapy (deceptive information)	Placebo
Antibiotic in a patient with suspected viral infection	Probably not indicated	Specific therapy	Placebo
Homoeopathic remedy in a child with a cold (prescribed by a sceptic but uncertain physician)	(Probably) placebo	Specific therapy	Placebo
Homoeopathic remedy in a child with a cold (prescribed by a homoeopath)	Specific therapy	Specific therapy	Placebo
Arthroscopic debridement in a patient with osteoarthritis of the knee	Specific therapy	Specific therapy	Placebo

Source: Linde K, Fässler M, Meissner K. Placebo interventions, placebo effects and clinical practice. Philos Trans R Soc Lond B Biol Sci. 2011;366(1572):1905-12.

In the diverse landscape of placebo use, five distinct scenarios demonstrate the complexity and variability in the application and understanding of placebos in clinical settings. The first scenario involves a pure placebo—a saline injection given to a pain patient under deceptive pretenses, making the patient believe it is a therapeutic treatment, although both the provider and the scientist acknowledge it as a placebo.^(27,28)^ This pure placebo usage has been reported by a significant percentage of healthcare professionals, typically aimed at benefiting the patient’s well-being or managing difficult patient interactions.^(2,29-31)^ Examples range from attempting to prevent drug dependency by substituting morphine with placebo injections to administering placebos to maintain a patient’s hope in the face of incurable conditions, occupying a grey area between ethical justification and the compromise of patient autonomy.

The remaining scenarios extend from the use of antibiotics in viral infections where they serve no real therapeutic purpose, considered an impure placebo, to the administration of homeopathic remedies perceived by some practitioners as effective despite the lack of active substances.^(32-34)^ Particularly contentious is the use of interventions like arthroscopic debridement in osteoarthritis, which some scientists categorize as a placebo despite being an invasive procedure believed by surgeons to be effective.^(35)^ These cases illustrate not only the blurred lines between genuine treatment and placebo but also highlight the disparity between scientific evidence and clinical practice. This begs the question: could all legitimate studies on the neuroscience of the placebo effect also be categorized within these scenarios? If so, this warrants a critical reassessment of the semantics surrounding placebo interventions, ensuring that the terminology and its implications are aligned with both ethical standards and scientific evidence.

All these paradoxes emerge because placebos are inert substances.^(36)^ In semantic debates, Grünbaum has proposed divisions such as non-placebo, for therapies with proven effects on a specific problem; generic placebo, which is further divided into intentional placebo and inadvertent placebo. In intentional placebo, the healthcare professional knows there is no proven effect but believes that incidental characteristics of the treatment might benefit the patient and may occasionally induce the patient to believe that the substance without proven effect could be beneficial; and inadvertent, where neither the professional nor the patient knows it is a substance without effect.^(37)^

In clinical research, placebo control groups are implemented primarily to guard against the logical fallacy of *post hoc, ergo propter hoc*—assuming that because one event follows another, the first event caused the second.^(38)^ This principle is a cornerstone of logical reasoning and scientific methodology and is intended to distinguish between the natural progression of disease and the actual impact of the intervention. Consequently, in semantic discussions, some scientists have proposed that the term “placebo” be used only in clinical trial settings.^(39)^

Nevertheless, the interpretation of outcomes in clinical trials is often prey to the very fallacy that such trials are designed to avoid. Many studies and practitioners attribute observed changes within a placebo group to the placebo itself, without considering a multitude of factors that could influence these changes, such as the natural progression of the disease, regression to the mean, or other unrelated variables.^(40)^ This misinterpretation blurs the lines between correlation and causation, suggesting that improvements or changes after administering an inert substance were caused by the substance itself, which is a critical issue in the ongoing debate about placebo effects.^(41)^

It is a categorical error to interpret changes in the control arm of a randomized trial as evidence that an inert substance has exerted a therapeutic effect.^(42^ By design, the placebo comparator lacks specific pharmacologic action for the condition under study. Any within-arm change after administration of an inert substance therefore reflects a composite of natural history, regression to the mean, measurement variability, co-interventions, and contextual effects—not a causal effect of the inert object itself. Causal effects can only be inferred from prespecified between-arm contrasts, not from changes over time within the placebo arm (Figure 1).

**Figure 1:**
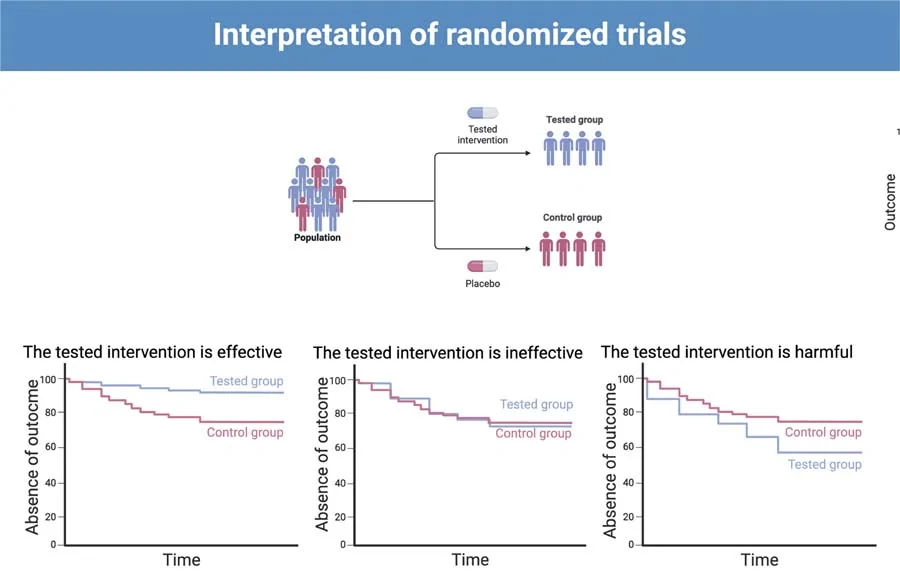
Possible outcomes of a randomized controlled trial

The ‘t+ested group’ receives the active treatment, while the ‘Control group’ receives a placebo. The first scenario shows the tested intervention to be effective, where the tested group exhibits a lower incidence of outcomes over time compared to the control. The second scenario demonstrates the tested intervention to be ineffective, with both groups showing parallel outcomes. The third scenario depicts the tested intervention as harmful, with the tested group having a higher incidence of outcomes than the control group. Importantly, none of these scenarios is interpreted in terms of the placebo; rather, the interpretation always concerns the intervention being tested.

As a result of these misnomers and misinterpretations, the concept of a placebo effect has gradually expanded far beyond its original scope and now encompasses a wide range of beneficial effects attributed to various biological, social, or human interactions that do not involve pharmacologically active drugs. This broad use of the term distorts its meaning and applies it to almost any positive change observed after an intervention, irrespective of the mechanism behind it. This interpretation of placebo effects includes improvements in patient conditions that are likely attributable to factors such as the natural progression of the disease, the patient’s expectations, the therapeutic environment, or even the care provided by healthcare professionals, rather than to inert substance, or placebo, itself.^(43)^

This expansion of the placebo effect’s definition risks creating a dangerous gap through which treatments of questionable efficacy can be misleadingly marketed as having substantial therapeutic benefits. Rather than openly admitting that such interventions are mere placebos, quacks and charlatans capitalize on this ambiguity, justifying their unconventional and often bizarre procedures by claiming they trigger real biochemical pathways and neural networks—assertions to which science has lent credence to. This misrepresentation is troubling as it can mislead patients away from proven, evidence-based medical treatments by offering false assurances of effectiveness.^(44)^ Fabrizio Benedetti, a prominent researcher in the neuroscience of placebo, admits that even honest science research has unconsciously produced a shift in the thinking of advocates of quackery. ^(44)^

According to Nunn, the persistent ambiguities surrounding the concept of the placebo are beyond resolution since every perspective on placebos attracts valid critique for failing to provide clarity.^(45)^ He contends that the inherent difficulties in defining the placebo effect call into question its very existence. If a concept remains indefinable and nonsensical from all angles, it prompts a reevaluation of its actuality. Nunn posits that there is no tangible entity corresponding to ‘placebo’ that naturally exists; thus, the term does not correspond to any distinct phenomenon in nature. Upon examining the wide array of treatments labeled as ‘placebos,’ it becomes challenging to identify any common characteristics they share. Nunn proposes that the discourse of medicine would be significantly clearer and more precise if discussions about placebos were abandoned entirely.^(45)^

However, given the significant advancements in neuroscience related to the neuropsychological effects of context and expectations, Nunn’s stance might appear somewhat exaggerated. A viable solution to this conceptual dilemma could be to adopt the term “contextual effects,” as proposed by Di Blasi et al^(46)^ This nomenclature acknowledges the substantial influence of contextual factors and patient expectations on therapeutic outcomes, distancing the discussion from the problematic ambiguities associated with the placebo term. Moreover, the term “placebo” used to describe “the effect of the patient-provider interaction” is not uncommon in the literature or colloquially in medical settings. In this context, the placebo effect is the culmination of all psychological processes that occur during the doctor-patient interaction. A typical example of an effect resulting from patient-provider interaction is a case in which a patient experiences a reduction in pain following a consultation with an empathic physician.^(47)^ For this reason, the phrase placebo effect should not be used as a causal label. In the next section, we distinguish the null effect of inert comparators from contextual effects and propose a nomenclature that prevents these misattributions.

## FACTORS THAT INFLUENCE THE PERCEPTION OF TREATMENTS

The assumption that inert substances, by themselves, may improve the condition of a disease ignores the fundamental principle that spontaneous health improvement can arise from several other factors:^(48)^

- Spontaneous improvement and natural disease progression: Diseases often have a natural history of fluctuating symptoms in which there may be a period of improvement without any medical intervention. This natural variability is particularly apparent in chronic diseases or conditions involving chronic pain, in which symptoms may vary significantly from day to day. Observing a group of patients with chronic diseases will invariably reveal that some individuals have experienced temporary improvements, creating the illusion of a placebo effect.^(49)^
- Co-intervention effects: The so-called placebo effect is often observed in the context of other treatments. The labelling of these improvements as a placebo effect is misleading. For example, in a study on angina pectoris included in Beecher’s analysis, the placebo group also received nitrates, which are known to be an effective treatment for this condition.^(39)^
- Unidentified co-interventions: Participation in a trial can increase patients’ awareness of their condition, leading to behavioral changes that may positively affect their health. For example, hypertensive patients may reduce their salt intake, minimize stress, or make other lifestyle changes after enrolling in a study. Although these changes are beneficial, they should not be confused with a placebo effect, since they are independent actions taken by the patient.^(50)^
- Observational Biases: The Hawthorne effect, an observational bias where individuals modify their behavior because they know they are being observed, can further contribute to the misconception of placebo efficacy.^(51)^
- Contextual and Neuropsychological Effects: Advances in neuroscience have highlighted the significant role of contextual and neuropsychological factors in shaping treatment outcomes. The patient’s environment, combined with their psychological and emotional state,^(52)^ can substantially influence the perception and experience of symptoms and therapeutic responses.

## A PROPOSED NOMENCLATURE

To resolve the persistent ambiguity surrounding the term placebo effect, we propose a nomenclature that separates three distinct elements: (i) the null effect of an inert comparator, (ii) contextual effects mediated by expectations and meaning, and (iii) deceptive inert- substance use in clinical practice. Our objective is not semantic refinement for its own sake but clarification of causal attribution. The proposed terminology acknowledges that meaningful outcomes can arise from therapeutic context while avoiding the scientifically incorrect implication that inert substances exert specific physiologic effects.

Before detailing our terminology, it is important to clarify that our use of the term inert refers specifically to the absence of direct, specific pharmacological activity for the condition being treated. We do not deny that inert substances—particularly within a therapeutic context—can produce meaningful physiological or psychological outcomes. However, these outcomes, which stem from the clinical environment, patient expectations, and symbolic meaning, are contextually mediated and not attributable to the intrinsic properties of the substance itself. This distinction is critical to avoid conflating the effects of context with the efficacy of an inactive agent.

This reclassification is essential to differentiate the legitimate scientific roles of placebos in clinical research from their misuse in deceptive practices:

- Null effect of an inert substance: Refers to the absence of specific pharmacological action from a substance administered in a clinical trial or therapeutic context – i.e., the baseline effect of an inactive agent.

- Placebo: Reserved exclusively for inert substances used within RCTs to minimize bias and isolate the effect of the tested intervention.

- Contextual or Neuropsychological Effects: Denotes therapeutic outcomes arising from non-pharmacological factors, primarily mediated by central nervous system processes such as expectation, emotional regulation, and symbolic meaning. These effects are triggered by cues from the clinical environment, healthcare interactions, and treatment rituals. While “contextual effects” alone might broadly suffice, we retain the term “neuropsychological” to highlight top-down mechanisms involving cortical and subcortical modulation of both subjective symptoms and objective physiological outcomes – including effects often labeled as ‘placebo responses.’

- Placebo effect: A paradoxical and ambiguous term that should be avoided. It conflates distinct phenomena – contextual modulation, natural disease progression, and statistical artifacts – with pharmacodynamic action from an inert agent, thereby risking misleading interpretations.

- Deceptive Placebo use: Refers to the unethical use of treatments lacking scientific evidence of efficacy, administered with either intentional deception or epistemic ignorance. In both cases, the practice undermines informed consent and violates ethical standards, especially when presented as effective outside controlled research settings.

Incorporating these terms, we revisit the scenarios previously presented in table 1, as detailed in table 2. This nomenclature may alleviate the paradoxes present in the current terminology, which have been exploited by quacks and charlatans.

**Table 2:** Clinical scenarios with revised nomenclature according to the proposed classifications of placebo, contextual effects, and deceptive placebo

Scenario	Proposed nomenclature
Saline injection in a patientwith pain	Deceptive Placebo
Antibiotic in a patient with suspected viral infection	Deceptive Placebo
Homeopathic remedy in a child with a cold (prescribed by a sceptic but uncertain physician)	Deceptive Placebo
Homeopathic remedy in a child with a cold (prescribed by a homoeopath)	Deceptive Placebo
Arthroscopic debridement in a patient with knee osteoarthritis	Deceptive Placebo
Effects of a positive doctor-patient relationship without intent to deceive, based on genuine neuropsychological responses to therapeutic contexts	Contextual or Neuropsychological Effects
Placebo pill administered to a research participant	Placebo

## METHODS THAT ESTIMATE CONTEXTUAL EFFECTS

If one aims to prove that the relative risk (RR) of an inert substance in a study differs from what would be expected based on the natural history of the disease, regression to the mean, co-interventions, and observational biases, and that there is, in fact, a contextual effect in that strategy, it is necessary to conduct a study or, if this was not done in the first place, to propose a new study with the following methodology: Placebo 1, which in this new study is referred to as an “Intervention,” versus Placebo 2, another inert substance with different components. ^(53,54)^ If a difference in relative risk between the two groups is observed after follow-up, then Placebo 1 can indeed be considered beneficial for the disease and will no longer be termed a placebo. At this point, we assume that it would be ethical to market this substance as an intervention. It is paradoxical to think that if a placebo substance is placed on the market as an intervention, it would no longer be considered a placebo because it has demonstrated an effect and, thus, it is not inert for that condition. It distinguishes itself from a placebo, since, by definition, a placebo is, by definition, inert . In other words, even if the effect of a placebo has been validated by an appropriate methodology, it cannot be referred to as a placebo effect.

However, we recognize that such a difference in outcomes between Placebo 1 and Placebo 2 might not necessarily indicate the efficacy of Placebo 1, but rather a nocebo effect caused by Placebo 2. Since nocebo effects are also contextually mediated, the interpretation remains ambiguous. To address this, a third inert comparator would be necessary to verify whether the observed difference stems from a harmful contextual effect linked to Placebo 2 or from a beneficial contextual effect associated with Placebo 1. This recursive need for additional arms in trial design illustrates the complexity of attributing causal interpretations to inert substance outcomes and reinforces the need for methodological rigor and conceptual clarity when testing contextual effects.

Employing the open-hidden paradigm may also provide vital insights into whether the awareness of receiving a treatment influences outcomes.^(55)^ This method involves administering both an active treatment and a placebo in overt and covert manners.^(56)^ For instance, Benedetti et al.^(57)^ observed that while both saline and proglumide had no direct analgesic effect when administered covertly, their pain-reducing impact was significantly more pronounced when given openly, suggesting that overt administration potentiated an endogenous opioid system by . Another strategy may involve comparing adherents and nonadherents within the placebo arm when the nonplacebo control resembles the nonadherents’ experience^(13)^ (Figure 2). These methods demand careful adjustment for confounders to avoid biased results. In statistically robust reanalyses of classic studies on adherents and non-adherents, for example, logistic regression tends to show a loss of the beneficial effect originally published.^(58,59)^ When analyzing studies like these, we must understand that adherence or non-adherence should not be seen as random but rather as a characteristic of the subject that may be associated with various other characteristics which, in sum, could have led to a poorer outcome.

**Figure 2:**
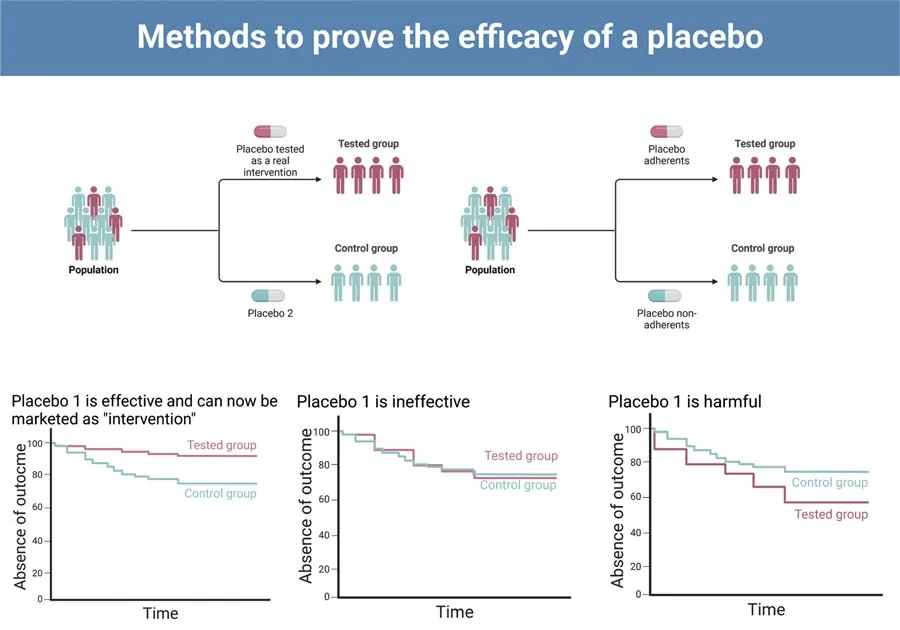
Methods used to estimate contextual effects and evaluate inert-substance interventions

This graph illustrates a comparative methodology evaluating ‘Placebo 1’ against ‘Placebo 2’, different inert substances, to ascertain therapeutic benefits. It also compares outcomes between placebo adherent and non-adherent participants to assess the impact of adherence. Outcomes show whether ‘Placebo 1’ is effective (fewer negative outcomes in the tested group), ineffective (no significant difference), or harmful (more negative outcomes). A significant relative risk difference suggests the therapeutic potential of ‘Placebo 1’, supporting its ethical consideration as a genuine intervention.

## THE ETHICAL PERSPECTIVE ON PLACEBOS

The term “placebo” derived from the Latin expression meaning “I will please” underscores a critical ethical dilemma in medical practice. Physicians are primarily tasked not to please, but to aid and heal.^(60)^ The so-called “duty to deceive”^(29)^ does not exist if the effect of a placebo, if clearly understood as the null effect of an inert substance, is nonexistent. It is regrettable that there are cases where the desire to provide more substantial assistance to patients exceeds the capacity of existing medical interventions. In such cases, it is an ethical imperative for physicians not to harm or, at the very least, to respect honesty in their interactions with patients.

The use of deception to provide reassurance, comfort, and hope is not necessary and overlooks the fundamental competences necessary for a physician’s relationship with patients. This includes taking time to understand the patient, promoting openness and active listening, removing obstacles to communication, allowing patients to share their experiences, sharing authority in decision-making processes, and demonstrating commitment. The “null effect of an inert substance” should not replace these core aspects of patient care.^(61)^ The effect of genuine care is present even when no deceptive substance is given.^(62)^

The use of a placebo as a quick solution for difficult patient encounters poses significant risks. It may lead to delays in the diagnosis of serious diseases and undermine the fundamental trust that forms the basis of the doctor-patient relationship.^(63)^ Misleading a patient constitutes a grave violation of this trust and can provoke significant negative repercussions.^(64)^ In addition, the use of placebos may perpetuate medical arrogance and contribute to the infantilization of patients, thereby treating them as less able to take rational care of their own healthcare.^(65)^

There are significant and sometimes substantial contextual or neuropsychological effects in human response to treatments. Such effects involve symptom relief primarily through the brain’s natural mechanisms, activated by the therapeutic environment, rather than by the inert substance itself^(4)^. It is implausible – and perhaps unethical – to disregard these contextual effects as irrelevant to health and pathology, given their demonstrated impact on symptoms like pain, depression, and fatigue.^(52)^

According to Braillon, there is no need for a complex debate on ethical considerations related to deceptive placebos. The guiding question should be: “Whose interests are being served?” In addition to the need to provide effective treatments based on the best available evidence, the ethical challenges associated with the use of placebo are highlighted.^(60)^ The priority should always be to serve the best interests of the patient and to comply with evidence-based practices that actually improve health outcomes.

In research settings, the use of “placebo” should remain strictly confined to its role within controlled clinical trials to distinguish its effects accurately from those of active treatments.^(66)^ At the same time, the application of placebos, or what may be better termed “contextual or neuropsychological effects,” in clinical practice must be approached with caution, ensuring it does not cross into deceptive conduct. The Declaration of Helsinki and other ethical guidelines reinforce the necessity of evaluating new therapies in relation to the standard of care, not merely against inert substances, thereby highlighting the limitations and potential harms associated with deceptive practices.^(67)^

While open-label placebos (OLPs) offer a strategy to preserve transparency and avoid deception, we argue that their effects are best conceptualized within the framework of contextual effects. The therapeutic benefit in OLPs is likely driven by the surrounding psychosocial context, patient expectations, and provider interaction, rather than the inert substance itself. In this sense, OLPs do not constitute a pharmacological intervention and should not be labeled as such. Their use may be ethically acceptable under rigorous trial conditions, but their routine clinical use requires caution and consistent empirical validation.

## CONCLUSION

Our thesis is that the placebo effect should be abandoned as a causal label in clinical care and research reporting. Outcomes observed after administration of an inert comparator should instead be partitioned into contextual effects and noncausal contributors such as natural history and regression to the mean. It has become evident that what has been traditionally termed the “placebo effect” encompasses a variety of phenomena, from the null effects of inert substances in controlled settings to the profound contextual or neuropsychological effects that occur in therapeutic environments. This analysis underscores the necessity of differentiating between mere inert substance effects and the meaningful therapeutic outcomes facilitated by the broader context of patient care, thereby advocating for a more precise and informed use of nomenclature in both clinical practice and research.
